# The Role of Immunohistochemical Markers for the Diagnosis and Prognosis of Adrenocortical Neoplasms

**DOI:** 10.3390/jpm11030208

**Published:** 2021-03-15

**Authors:** Anna Angelousi, Georgios Kyriakopoulos, Fani Athanasouli, Anastasia Dimitriadi, Eva Kassi, Chrysanthi Aggeli, George Zografos, Gregory Kaltsas

**Affiliations:** 1 Unit of Endocrinology, First Department of Internal Medicine, Laiko Hospital, National and Kapodistrian University of Athens, 11527 Athens, Greece; fani.athanasouli@yahoo.gr; 2 Department of Pathology, Evaggelismos Hospital, 11521 Athens, Greece; geokyr11@hotmail.gr; 3 Department of Surgical Pathology, General Hospital of Athens “G. Gennimatas”, 11527 Athens, Greece; dimitra_anastasia@yahoo.gr; 4 First Department of Propaedeutic Internal Medicine, Laiko Hospital, National & Kapodistrian University of Athens, 11527 Athens, Greece; ekassi@med.uoa.gr (E.K.); gkaltsas@endo.gr (G.K.); 5 Third Surgical Department of Surgery, General Hospital of Athens “G. Gennimatas”, 11527 Athens, Greece; chraggeli@yahoo.gr (C.A.); gnzografos@yahoo.com (G.Z.)

**Keywords:** adrenocortical cancer, adrenal adenomas, adrenal tumors, p53, p27, ki-67, reticulin

## Abstract

Adrenal cortical carcinoma (ACC) is a rare cancer with poor prognosis that needs to be distinguished from adrenocortical adenomas (ACAs). Although, the recently developed transcriptome analysis seems to be a reliable tool for the differential diagnosis of adrenocortical neoplasms, it is not widely available in clinical practice. We aim to evaluate histological and immunohistochemical markers for the distinction of ACCs from ACAs along with assessing their prognostic role. Clinical data were retrospectively analyzed from 37 patients; 24 archived, formalin-fixed, and paraffin-embedded ACC samples underwent histochemical analysis of reticulin and immunohistochemical analysis of p27, p53, Ki-67 markers and were compared with 13 ACA samples. Weiss and Helsinki scores were also considered. Kaplan−Meier and univariate Cox regression methods were implemented to identify prognostic effects. Altered reticulin pattern, Ki-67% labelling index and overexpression of p53 protein were found to be useful histopathological markers for distinguishing ACAs from ACCs. Among the studied markers, only pathological p53 nuclear protein expression was found to reach statistically significant association with poor survival and development of metastases, although in a small series of patients. In conclusion, altered reticulin pattern and p53/Ki-67 expression are useful markers for distinguishing ACCs from ACAs. Immunohistopathology alone cannot discriminate ACCs with different prognosis and it should be combined with morphological criteria and transcriptome analysis.

## 1. Introduction

Adrenocortical carcinoma (ACC) is a highly aggressive malignancy with an estimated worldwide prevalence of 4–12 cases per million adults and a five-year survival rate ranging from 16 to 38% [1,2]. Although several different scoring systems have been proposed to assess the malignant potential in adrenocortical neoplasms, the Weiss score remains the most utilized tool in distinguishing benign from malignant adrenocortical neoplasms [3]. This score counts nine histopathologic criteria: eosinophilic (“dark”) cytoplasm in more than 75% of tumor cells, a “patternless” diffuse architecture, necrosis, nuclear atypia, mitotic index above 5 per 50 high-power fields, atypical mitoses, sinusoidal, venous, and capsular invasion [4]. An adrenocortical neoplasm is classified as malignant when it meets three or more of these criteria [5]. However, the distinction of noninvasive low-grade ACC with a low Weiss score from adrenocortical adenoma (ACA) poses a diagnostic challenge especially in small-sized and purely localized lesions and in large tumors without invasive features or cellular atypia in which well-differentiated cells resemble those seen in ACAs [2,6].

In addition, intratumoral morphologic, proliferative, and molecular heterogeneity have been recognized in these adrenocortical neoplasms. Microscopic regions with low-grade proliferative features can be encountered in high-grade ACCs, and low-grade ACCs can contain areas indistinguishable from ACAs [7,8]. Furthermore, recent observations also suggest the possibility of adenoma−carcinoma progression in some adenomas although this needs to be confirmed [9,10,11,12]. In the last decade there has been enormous progress in our understanding of the molecular biology of adrenocortical neoplasms [13,14,15]. The development of genomics has led to a new classification of ACC by two independent international cohorts; one from the European Network for the Study of Adrenal Tumors (ENSAT) network [14] in Europe and the other from the Cancer Genome Atlas [8] consortium in America, Europe and Australia, with two distinct molecular subgroups, C1A and C1B associated with poor (5-year survival rate of 20%) and good prognosis (5-year survival rate of 91%), respectively [7,16,17]. The C1B group is characterized by low mutation rate, and a very low incidence of mutations of the main driver genes of ACC whereas the C1A group is characterized by high mutation rate and driver gene alterations. This group is further divided into a subgroup of aggressive tumors showing hypermethylation at the level of the CpG islands located in the promoter of genes (“CIMP phenotype”).

However, this transcriptome analysis is still not widely available making it necessary to utilize currently readily available histochemical and immunohistochemical markers for the distinction of adrenocortical neoplasms and their prognosis.

Several immunohistochemical markers have been proposed to improve the histological recognition of malignancy and eventually obtain a more precise characterization of these histologically characterized “grey zones” [16,18,19,20]. In order to provide prognostic biomarkers for the evaluation of surgical samples with adrenocortical neoplasms, we investigated the role of altered reticulin framework, a fast and cheap technique with high interobserver reproducibility, as well as of proteins involved in cell proliferation and mitotic spindle regulation such as Ki-67, p53, and p27 in a surgical series of benign and malignant adrenocortical neoplasms. We also studied their association with the clinical prognosis of patients with ACCs including progression-free survival (PFS) and overall survival (OS).

## 2. Materials and Methods

### 2.1. Samples and Clinicopathologic Parameters

We identified 65 consecutively treated patients with histologically confirmed ACCs (*n* = 35) and ACAs (*n* = 30) from the Endocrine Unit of the Laiko General Hospital. Paraffin-embedded blocks were available for 37 of these patients (24 ACCs and 13 ACAs). All surgical samples were reviewed by two experienced pathologists blinded to clinical history or outcome. The protocol of this study was approved by the institutional Research Ethics Board of the National and Kapodistrian University of Athens.

In all cases investigated, three consecutive 4 μm thick tissue sections were obtained from a representative neutral buffered formalin-fixed, paraffin-embedded sample. ACAs and ACCs were defined grossly and microscopically following the criteria and the nomenclature system of pathological features proposed by Weiss et al. [3]. All primary malignant adrenal tumors reviewed as part of this study demonstrated three or more of the histopathologic criteria needed for the diagnosis of ACC [3].

Markers of adrenal cortical differentiation (Steroidogenic Factor 1 (SF)-1, Melan-A, calretinin, alpha-inhibin, and synaptophysin) were applied at the time of the diagnostic workup of each neoplasm. All adrenal cortical neoplasms were classified according to the universal diagnostic criteria endorsed by the WHO classifications including the modified Weiss criteria as well as the Lin−Weiss−Bisceglia criteria [21,22]. The mitotic grade was assessed based on mitotic count in 50 high power fields (HPF) from high mitotic density areas in all samples. ACCs displaying up to 20 mitotic figures per 50 HPF were classified as low-grade carcinomas, whereas those exceeding 20 mitotic figures per 50 HPF were recorded as high-grade carcinomas [23,24]. Vascular invasion was defined by tumor cells invading through a vessel wall and/or intravascular tumor cells admixed with thrombus was recorded in all ACCs.

The available follow-up clinical information was reviewed to determine the status of disease including relapse and mortality rate, distant metastasis, PFS and OS.

### 2.2. Histochemistry and Immunohistochemistry

Each section series was stained with different methods:

Hematoxylin-Eosin (HE) to confirm the diagnosis of adrenal nodular lesions.

Monoclonal antibodies against Ki-67 (clone MIB-1, DAKO), p53 (Mouse clone DO-7, DAKO) and p27 (Mouse clone SX53G8, DAKO).

Formalin-fixed paraffin-embedded tissue sections (4 μm) were dewaxed in 5 changes of xylene and rehydrated through graded alcohols. Negative and positive control tissues were selected based on manufacturer recommendations (p27) as well as previous publications where these antibodies were applied (p53 and Ki-67%) [25]. Multiple control experiments were undertaken to optimize each antibody. Endogenous peroxidase was blocked with 3% hydrogen peroxide. For the p27 (clone SX53G8, DAKO) and p53 (clone DO-7, DAKO) immunohistochemistry the BOND Polymer Refine Detection System (Leica Biosystems) was used which contains a peroxide block, postprimary, polymer reagent, DAB chromogen and hematoxylin counterstain all ready-to-use for the automated BOND system. The Ki-67 (clone MIB-1, DAKO) immunohistochemistry was performed in an automated stainer (Ventana Benchmark).

Tissue microarray assays (TMA) blocks were subjected to Gordon-Sweet Silver histochemistry in order to reveal the reticulin framework in all tumors. The loss of reticulin network was scored as follows: score 1: no loss of reticulin framework; score 2: minimal loss ( <25%) of reticulin framework; score 3: focal loss (25% to 50%) of reticulin framework; and score 4: obvious loss ( >50%) of reticulin framework. Qualitative pattern changes on the reticulin framework were also documented [26].

For the evaluation of the Ki -67 proliferation index we calculated the percentage of positive cells by manual count of the hot spot area which always contained at least 500 cells. Weakly stained nuclei were also counted [27].

For the evaluation of p53 the recently suggested tripartite interpretation guide was used where an “overexpressed or no expression (all or nothing)” nuclear staining pattern was highly predictive of an underlying TP53 mutation while a normal/wild type pattern was not. The distribution of nuclear staining in a “wild type” pattern ranged from a few positive cells to almost all (“high” wild type staining due to high proliferation) cells staining, but with variable intensity with a few nuclei stained strongly. Overexpression defined as nuclear staining in at least 50% of tumor cell nuclei while overexpression in at least >80% was considered strongly associated with TP53 mutations [28,29].

For p27/kip-1 protein there was a qualitative and quantitative evaluation of the nuclear expression of the protein (analyzed by the pathologists) in the tumor cells where the intensity of the expression was determined as 0 (no expression), 1 (weak expression), 2 (moderate expression) and 3 (strong expression) and the percentage of tumor cells expressed p27/kip-1 was scored as 0 (<5%), 1 (5–25%), 2 (26–50%), 3 (51–75%), 4 (76–100%). The percentage of positive nuclei of cells was calculated in more than 1000 cells of five successive and representative high-power fields (×400 magnification microscope). The immunoreactive score (IRS) was applied to determine the final staining score by multiplication of the intensity score and the distribution score [30].

### 2.3. Statistical Analysis

All the data are reported as median (range) for continuous parameters and proportions for categorical variables. Differences between patients with ACCs and ACAs were assessed using Mann−Whitney test for continuous variables and Fisher’s exact test for categorical variables. For correlation analysis we used Spearman’s rank correlation coefficient test. The diagnostic accuracy of the markers was evaluated using the receiver operating characteristic (ROC) curve. The area under the ROC curve (AUC) was used to measure how well a marker can distinguish ACCs and ACAs. Based on the AUC, the test was considered excellent between 0.90 and 1.00; good between 0.80 and 0.90; fair between 0.70 and 0.80; and poor between 0.60 and 0.70, and the test was considered to have failed if the value was below 0.60 [31]. The Kaplan–Meier method was used to evaluate the PFS and survival of ACC patients. Univariate Cox proportional hazards models were used to examine the association between histopathological and immunohistochemical characteristics and the main end points of our study (PFS and OS). No multivariate analyses were performed because of the small number of cases. Statistical significance was set at 0.05 and all computation were made using PRISM 7.

## 3. Results

### 3.1. Clinical and Morphological Features of ACC and ACA Patients

In this study, we retrospectively examined 24 ACCs (median size = 10 cm, range 1–24 cm) and 13 ACAs (median size = 5 cm, range 1.3–9 cm) samples obtained from the archival files of 37 patients submitted to adrenal surgery (Table 1). The median age was 54.5 (21–76) and 63.5 (38–71) years for the 24 ACCs (14 females) and the 13 ACAs (9 females) patients, respectively. The 45.8% of the ACCs (3 cortisol-secreting, 2 aldosterone-secreting, 6 both cortisol and androgen secreting) compared to 61.5% of the ACAs (7 cortisol-secreting, 1 aldosterone-secreting) were functional.

Nine ACCs were classified as ENSAT stage 1–2 and 15 ACC as ENSAT stage 3–4. Five out of 24 ACC patients (20.8%) exhibited in computed (CT) or magnetic resonance (MRI) imaging distant metastases at diagnosis (2 patients had pulmonary, one liver, one both pulmonary and liver metastases and the last atrial thrombus) (Table 2).

Eight out of 24 ACC patients died (mortality rate 33.3%) at a median time of 18.4 (2.13–101.9) months from diagnosis. Thirteen (54.2%) patients relapsed or developed disease progression with a median PFS of 6.57(1.93–19.7) months during a median follow-up of 18.4 months (Table 2). Functionality in ACC patients was significantly associated with relapse (*p* = 0.001) and increased mortality (*p* = 0.043) (Table 3). All patients were treated with mitotane that was initiated either immediate after surgery for ENSAT stages 3,4 (*n* = 13) and for stages 1,2 (*n* = 5) or during relapse (for stages 3,4, (*n* = 3) and for stages 1,2, ( *n* = 3). The Kaplan−Meier survival curve for PFS and OS of all patients with ACCs is shown in (Figures S1 and S2). Univariate Cox regression analysis showed a significant association between PFS and functionality (*p* = 0.003), and PFS and ENSAT Stage (*p* = 0.047).

### 3.2. Expression of Histochemical and Immunohistochemical Markers in Adrenocortical Neoplasms

All ACC samples possessed 3 or more of the Weiss criteria (median score 6, ranging from 4 to 9) compared to 0 (median score 0, ranging 0–1) for all ACAs. Median Helsinki score was 28 (10–56) for ACCs and 3 (1–5) for ACAs (*p* < 0.001) samples (Table 1). The Weiss and Helsinki scores had statistically significant positive correlation (*r*^2^ = 0.33, *p* = 0.007). Neither Weiss nor Helsinki were found to be significantly associated with PFS or OS.

**Figure 1:**
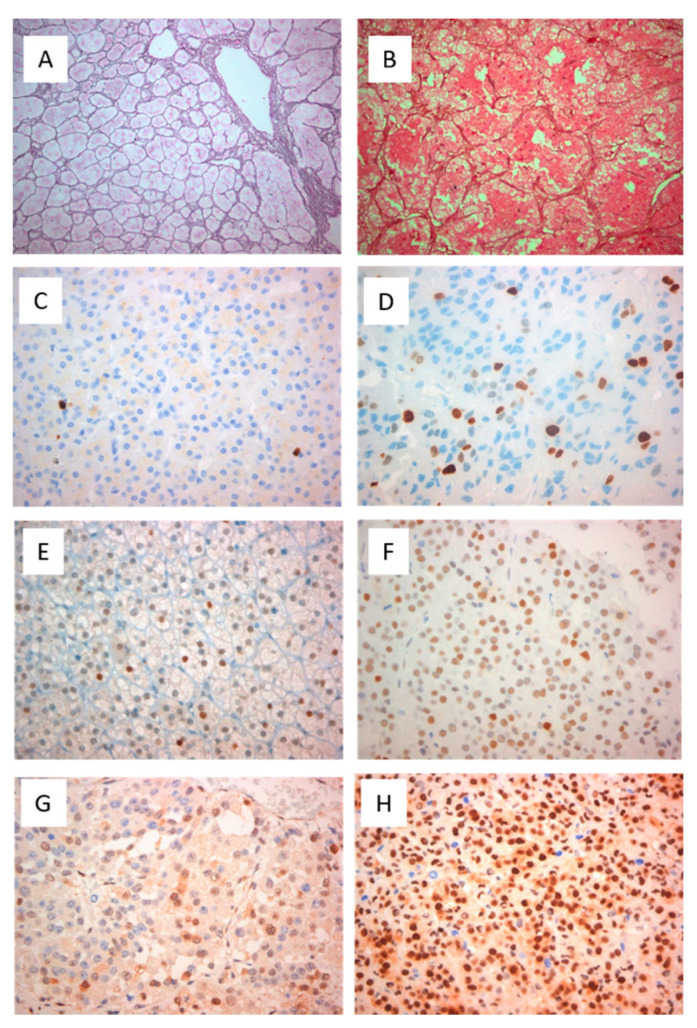
Histochemical and immunohistochemical expression of reticulin, Ki-67%, p53 and p27 in adrenocortical neoplasms. Intact reticulin framework with the characteristic acinar pattern in adrenocortical adenoma (ACA) (×200) (**A**). Highly disrupted architecture and loss of the reticulin framework in adrenocortical carcinoma ( ACC )(×400) (**B**). Increase of positive (brown stained) nuclei in Ki-67 immunostaining in ACA (**C**) compared to ACC (**D**). Strong nuclear expression (brown stained nuclei) of p53 protein in a few tumor cells in ACA (wild type pattern) (×400) (**E**), Strong nuclear expression of p53 protein in >80% of tumor cells in ACC (overexpression) (×400) (**F**). Moderate nuclear staining of p27 protein in ACA (×400) (**G**). Strong nuclear staining of p27 protein in the majority of cells in ACC (×400) (**H**).

The median reticulin scores were 4(3–4) and 0(0–1) for ACC and ACA samples, respectively, (*p* < 0.001) (Table 1). Eight out of 13 (62%) ACA samples displayed an intact reticulin framework (median score:0) (Figure 1A) whereas only 5 (38%) ACA samples presented a very focal minimal loss of reticulin (median score:1) (Figure 1B). All ACCs studied (18 samples) had an abnormal reticulin framework; 9 (50%) ACC samples showed highly disrupted architecture of the pericellular reticulin pattern (median score:3) whereas the other 9 ACC (50%) samples had a complete loss of reticulin pattern (median score: 4).

The nuclear expression of the proliferation marker Ki-67 labeling index was significantly higher for ACC compared with ACA samples (23.5% (15–45 for ACC vs. 3% (1–5) for ACA (*p* = 0.001)) (Table 1, Figure 1C). The cut off of Ki-67 > 5% exhibited a 92.3% sensitivity and 95.4% specificity for the distinction of ACC from ACA samples with high accuracy (AUC = 99%, *p* = 0.009).

Abnormal p53 protein expression in immunostaining was noted in 88.2% (15/17) of all ACC samples whereas wild type expression was described in only 11.8% (2/17) of them. All ACA samples showed wild type expression of p53 (*p* < 0.001) (Table 1, Figure 1E). Nine patients (8 with pathological expression of p53) had stage 3 or 4 and 8 (7 with pathological expression of p53) had stage 1or 2.

**Figure 2:**
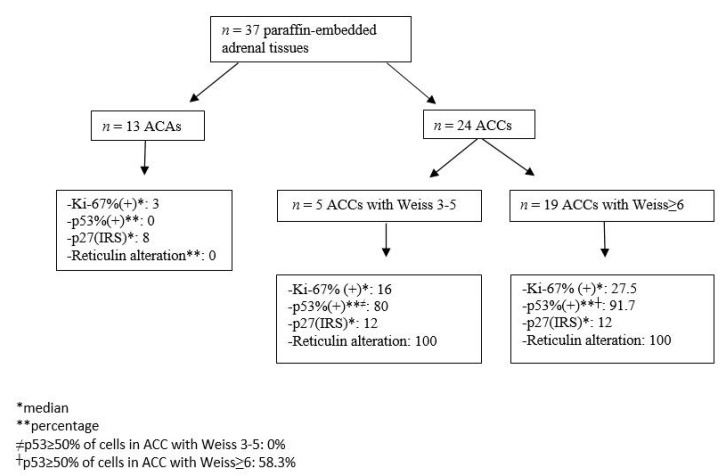
Expression of the Ki-67%, p53, p27 immunohistochemical nuclear markers as well as reticulin expression in adrenocortical neoplasms. All ACA cases exhibited Ki-67% labeling index ≤ 5% (median: 3%) and expressed the wild type p53 expression. No altered reticulin pattern was observed and median IRIS of p27% staining was 8. In all ACC cases Weiss was higher than 3, with altered reticulin pattern and similar IRS of p27 staining. However, those with relatively lower Weiss 3–5, exhibited lower median Ki-67% labeling index and p53 staining compared with ACC cases with Weiss scores greater than 6. Abbreviations: ACA: adrenocortical adenoma; ACC: adrenal cortical carcinoma; IRS: immunoreactive score; WT: wild type

Eight ACAs showed moderate p27 expression and 5 strong whereas in ACCs all but 2 showed strong expression (Figure 1G–H). No statistically significant differences in the expression of the markers studied were observed concerning the secretory component of these neoplasms (functional versus nonfunctional). The schematic presentation of immunohistochemical markers’ expression in adrenocortical neoplasms is shown in Figure 2.

### 3.3. Prognostic Role of the Histopathological Markers in ACC Patients

All ACC samples with altered reticulin pattern had concomitant necrosis and/or capsular/vascular invasion and/or increased mitotic activity (>5/50 HPF). Thus, using the reticulin algorithm all ACCs would be diagnosed as malignant (significant positive correlation with Weiss score, *r*^2^ = 0.72 *p* < 0.0001). Ki-67 labeling index was positively associated with a mitotic count >5/50 HPF (*r*^2^ = 0.16, *p* = 0.049) and a Weiss score (*r*^2^ = 0.31, *p* = 0.007) but not with vascular or capsular invasion. The p53 expression was also significantly correlated with mitotic count (>5/50 HPF) (*p* = 0.02), but not with capsular or vascular invasion. No statistically significant difference was noted between high-grade and low-grade ACCs in respect to reticulin pattern, p27 staining (*p* = 0.366), p53 staining (*p* = 0.485) and Ki-67 labeling index (*p*= 0.074) although in the last case it reached statistical significance and high grade ACCs had higher median (min-max) Ki-67 labeling index (30% (15–45)) compared with low-grade ACCs (16% (15–45)).

Neither Ki-67 labeling index, p27 expression nor reticulin pattern score were found to be significantly associated with PFS or OS (Table S2). However, p53 abnormal expression (≥ 50% of positive cells) was statistically significantly higher in metastatic ACC samples compared to nonmetastatic ACC samples (*p* = 0.035) as well as in nonsurvivors compared to survivors (*p* < 0.001) (Table S1, Table 3). A cut off of 23% of the percentage of p53-immunostaining positive cells was significantly associated with PFS (AUC 82%, *p* = 0.0013, sensitivity = 71%, specificity 100%).

## 4. Discussion

In the current study, among the studied markers, altered reticulin pattern, Ki-67% labeling index value and abnormal nuclear expression of p53 protein were found to be statistically significant histopathological and molecular markers for distinguishing malignant from benign adrenal neoplasms although in a small series of patients. In contrast, p27 expression was not found to be a significant marker for the distinction of benign from malignant adrenal cortical neoplasms. However, only the pathological p53 nuclear protein expression was found to have a prognostic role since it was significantly correlated with mortality rate, PFS and metastatic status. The reticulin algorithm defines malignancy through an altered reticulin framework associated with one of the three following parameters: necrosis, high mitotic rate, and vascular invasion for the diagnosis of ACCs [18,19,32,33]. The “reticulin” diagnostic algorithm has been proposed, based on the observation that the tumoral reticulin framework (highlighted by reticulin silver-based histochemical staining) is consistently disrupted in malignant cases but only in a small subset of benign cases. Several case series on ACCs have confirmed the usefulness of the reticulin algorithm for the distinction of ACCs from ACAs [2,19,32,33,34], although without showing any correlation with prognosis in these patients [6,35]. Our series confirmed the quantitative loss of reticulin framework as a significant finding, distinguishing ACCs and ACAs, showing also a significant positive correlation with Weiss score without being significantly associated with prognosis.

Adrenal cortical malignancy is a proliferation-driven malignancy and this study demonstrated significantly increased expression levels of markers related to cell proliferation such as Ki-67 and p53 in ACCs compared with ACAs. Nuclear expression of the proliferative marker Ki-67 was also significantly correlated with mitotic activity and Weiss score. We have also demonstrated with high accuracy that a Ki-67 labeling index cut-off value > 5% (92.3% sensitivity and 95.4% specificity) could discriminate ACCs from ACAs, confirming the existing data of the literature [9,10,36]. However, this series failed to confirm the current literature and showed no statistically significant association of Ki-67% with PFS and OS, probably due to the small number of samples and the short follow-up period. In line with these data in the literature, there are also some studies that have not confirmed a significant association of Ki-67% with either OS [37] or disease-free survival [38] although in the last study Ki-67% was found to be an independent prognostic factor for OS. Moreover, although the practical utility of Ki-67% staining was indisputable and confirmed in many studies, there are also studies supporting the idea that it is hard to set a diagnostic threshold that is mainly attributed to possible interobserver variations [39].

The cell cycle regulation molecular marker p53 encoding a protein that promotes DNA repair, was present in almost all ACC (15/17) and absent in ACA samples. The half-life and expression of p53 protein is low and therefore undetectable by immunohistochemistry [6,40,41]. Aberrant nuclear immunohistochemical staining for p53 in ACC samples varies in the literature from 5% to 60% [42,43]. In adult sporadic ACCs, about one quarter of tumors harbor somatic TP53 mutations [43,44,45], and more than a half harbor loss of heterozygosity at the TP53 locus [13,46]. Previous studies, have shown that ACAs had significantly lower levels of immunohistochemical p53 nuclear expression than ACCs [6], whereas others have failed to show such a difference [47]. Moreover, it has also been reported that high-grade ACCs exhibit higher p53 expression than low-grade ones, a finding consistent with the enrichment of TP53 mutations in high grade carcinomas [9,48].

Transcriptome studies have led to further understanding of the role of p53 in sporadic ACCs. Indeed, TP53 mutated tumors are enriched in a subgroup of ACCs identified by unsupervised clustering of the tumors [17]. Finally, genes positively regulated by p53 such as RRM2B, TP53INP1 and MDM2, were found to be downregulated in this subgroup. The present series confirmed the aberrant nuclear immunohistochemical expression of p53 in ACCs compared to ACAs, although 11.8% of the ACCs showed wild type expression of p53 as all ACAs. Abnormal expression of p53 was the only marker in our series of adrenal neoplasms that showed to have a prognostic role since it was associated with increased mortality rate and the presence of metastases, whereas a cut off of 23% of the percentage of p53-immunostaining positive cells was significantly associated with PFS. Although in molecular analysis the prognostic role of the abnormal p53 expression in ACC is clear [17], immunohistochemical data on the prognostic role of p53 are rather conflicting. Several studies have failed to show a prognostic role of p53 protein [6,42,49,50], whereas others have shown that patients with abnormal p53 staining tended to have higher grade and stage ACCs tumors, increased relapse rates and poorer disease-free survival [46,51].

The protein encoded by p27 (CDKN1B) is another cell cycle regulator marker, that when it is upregulated, results in cell cycle arrest and apoptosis [52]. Our study found no statistical difference of p27 expression between ACCs and ACAs. Accordingly, two previous studies [50,53] have failed to show that p27 could be used as an immunohistochemical markers for distinguishing ACCs from ACAs. However, a more recent study showed that p27 staining was significantly higher in ACCs compared with ACAs, using an automated method of analysis with a high diagnostic accuracy of 7.23% as the best cut-off value [47]. This study had the novelty to use an automated method of analysis in contrast to previous studies which were carried out by direct observation by the researchers [50,53].

All ACC samples exhibited positive staining for Ki-67, p53 and p27 except for two ACC samples that were p53 negative and p27 and Ki-67 positive. In contrast to p53 staining which was found normal in all ACAs (wild type), Ki-67 and p27 were positive in all ACAs. The overexpression of p27 in ACC samples is somewhat contradictory. Several explanations have been proposed, either that adrenal cancer cells develop a tolerance to this inhibitor of cell cycle progression, suggesting that p27 could be present but inactive to arrest the cell cycle, or that p27 gene is mutated resulting in a modified p27 protein [47,54].

Our study has several limitations, that are mainly related to its retrospective nature and the number of samples analyzed. The relatively limited number of samples did not allow a more fruitful statistical analysis that, along with the short follow-up period, may have affected the identification of the prognostic role of the immunohistochemical biomarkers in these tumors. We anticipate that the inclusion of more patients may provide the additional power to reach meaningful clinical findings.

In conclusion, Ki-67, p53 as well as abnormal reticulin pattern, but not p27 expression, could be used to define malignancy in adrenocortical neoplasms and differentiate ACCs from ACAs. Furthermore, p53 expression was significantly associated with increased mortality, metastatic status and lower PFS. However, the small number of patients did not allow a more robust conclusion; perhaps if confirmed in larger studies it may offer a diagnostic/prognostic tool available in everyday clinical practice. Immunohistopathology alone cannot fully discriminate ACCs with poor prognosis from those with good prognosis and it should be combined with further morphological criteria and recently developed transcriptome analysis which have shown clear differences between adenomas and high- or low-grade carcinomas.

## Figures and Tables

**Table 1:** Comparisons of the clinical, histopathological and immunohistochemical characteristics of the adrenocortical neoplasms.

Characteristics	ACCs (*n* = 24)	ACAs (*n* = 13)	*p*-Value
Clinical characteristics			
Age (median(min−max)), ys	54.5(21–76)	63.5(38–71)	0.119
Sex (Female, *n*(%))	14(58.3)	9(69.2)	0.724
Size (median(min-max)), mm	10(1–24)	5(1.3–9)	0.002
Functionality (*n*(%))	11(45.8)	8(61.5)	0.495
Histopathological characteristics (median(min-max))			
Weiss	6(4–9)	0(0–1)	0.001
Helsinki	28(10–56)	3(1–5)	<0.001
Reticulin score	4(3–4)	0(0–1)	<0.001
Immunohistochemical characteristics			
Ki-67% (median (min-max))	23.5(15–45)	3(1–5)	<0.001
p27 (IRS) (median(min-max))	12(4–12)	8(8–12)	0.121
p53 (pathological, *n*(%))	15(88.2)	0(0)	<0.001
p53 (WT vs. overexpression 20–49% vs. overexpression ≥50%, *n*(%))	2(11.)/8(47.1)/7(41.2)	All wild	<0.001

Abbreviations: ACC: adrenal cortical carcinoma; ACA: adrenocortical adenoma; IRS: immunoreactive score; WT: wild type.

**Table 2:** Clinical and histopathological characteristics of ACC.

**Clinical Characteristics**	
Stage (median (min-max))	3 (1–4)
1 or 2 (*n*(%))	9 (37.5)
3 or 4 (*n* (%))	15 (62.5)
Metastatic at presentation (*n* (%))	5 (20.8)
Duration of mitotane treatment (median (min-max)), months	9.8 (4.6–36.9)
PFS (median (min-max)), months	6.57 (1.93–19.7)
Rate of PD (*n* (%))	13 (54.2)
Mortality (median (min-max)), months	11.4 (5.63–27.5)
Mortality rate (*n* (%))	8 (33.3)
Follow-up (median (min-max)), months	18.4 (2.13–101.9)
**Histopathological Characteristics**	
Capsular invasion (*n* (%))	19 (79.2)
Vascular invasion (*n* (%))	11 (45.8)
Necrosis (*n* (%))	23 (95.8)
Mitoses >20 per 50 HPF (*n* (%))	11 (45.8)
Atypical mitoses (*n* (%))	21 (87.5)

Abbreviations: ACC: adrenal cortical carcinoma; PFS: progression free survival; PD: progression disease; HPF: high-power fields.

**Table 3:** Association of clinical, histopathological and immunohistopathological markers with prognostic factors.

Characteristics	No Relapse	Relapse	*p*-Value	No Death	Death	*p*-Value
Clinical						
Size	9(3.5–15.2)	10(1–24)	0.417	9.5(3.5–24)	11(1–16)	0.759
Functionality (*n* (%))	1(9.09)	10(76.9)	0.001	5(31.3)	6(75)	0.043
Histopathological						
Weiss (median (min-max))	6(4–8)	6(4–9)	0.434	6(4–8)	6.5(6–9)	0.481
Helsinki (median (min-max))	30(10–56)	28(20–53)	0.619	28(10–56)	33(23–53)	0.426
Capsular invasion (*n* (%))	7(63.6)	12(92.3)	0.142	12(75)	7(87.5)	0.631
Vascular invasion (*n* (%))	4(36.4)	7(53.9)	0.444	6(37.5)	5(62.5)	0.390
Nuclear atypia (*n* (%))	10(90.9)	13(100)	0.458	15(93.8)	8(100)	0.999
Mitoses >20 per 50HPF (*n* (%))	3(27.3)	8(61.5)	0.123	7(43.8)	4(50)	0.999
Reticulin (score 4, *n* (%))	5(55.6)	4(44.4)	0.999	6(50)	3(50)	0.999
Immunohistopathological (median(min-max))						
Ki-67%	23.5(15–40)	25(15–45)	0.668	23.5(15–40)	25(15–45)	0.785
p27([IRS)	12(8–12)	9(4–12)	0.175	12(6–12)	9(4–12)	0.302
p53 (pathological, *n* (%))	7(87.5)	8(88.9)	0.999	9(81.8)	6(100)	0.515
p53 (WT/overexpression 21–50%/≥ 50%, *n* (%))	1(12.5)/6(75)/1(12.5)	1(11.1)/2(22.2)/6(66.7)	0.057	2(18.2)/8(72.7)/1(9.1)	0(0)/0(0)/6(100)	0.001

Abbreviations: ACC: adrenal cortical carcinoma; HPF: high-power fields; IRS: immunoreactive score; WT: wild type.

## Data Availability

The data presented in this study are available on request from the corresponding author. The data are not publicly available due to privacy restrictions.

## Supplementary Materials

The following are available online at https://www.mdpi.com/2075-4426/11/3/208/s1, Table S1: Association of clinical, histopathological and immunohistopathological markers with prognostic factors, Table S2: Univariate Cox regression analysis for risk factors associated with PFS and OS in 24 patients with ACC, Figure S1. Kaplan−Meier curve for PFS of patients with ACC, Figure S2. Kaplan−Meier curve for OS of patients with ACC.
