# Supplementary material for: The Role of Immunohistochemical Markers for the Diagnosis and Prognosis of Adrenocortical Neoplasms
Source: J Pers Med. 2021 Mar 15;11(3):208. doi: 10.3390/jpm11030208 (PMC8001501; doi:10.3390/jpm11030208)
Supplement: Supplementary file 1 [file jpm-11-00208-s001.pdf]

Table S1. Association of clinical, histopathological and immunohistopathological markers with prognostic factors

Characteristics	Stage 1-2	Stage 3-4	p-value	No Metastases at diagnosis	Metastases at diagnosis	p-value
Clinical						
-Size	9 (4-12)	12(1-24)	0.107	9(1-24)	13.2(9.5-17)	**0.039**
-Functionality [n (%)]	7 (53.9)	8(72.7)	0.423	2(15.4)	3(27.3)	0.630
Histopathological						
-Weiss [median (min-max)]	5(4-8)	7(6-9)	**0.005**	6(4-8)	6(6-9)	0.414
Helsinki [median (min-max)]	24(10-38)	33(20-56)	0.096	30(10-56)	25.5(23-48)	0.788
-Capsular invasion [n (%)]	5(55.6)	14(93.3)	**0.047**	14(73.7)	5(100)	0.544
-Vascular invasion [n (%)]	3(33.3)	8(53.3)	0.423	7(36.8)	4(80)	0.142
-Nuclear atypia [n (%)]	8(88.9)	15(100)	0.375	18(94.7)	5(100)	0.999
-Mitoses >20 per 50HPF [n (%)]	2(22.2)	9(60)	0.105	8(42.1)	3(60)	0.630
Reticulin [score 4, n (%)]	3(37.5)	6(60)	0.637	7(50)	2(50)	0.999
Immunohistopathological [median (min-max)]						
-Ki-67%	17(15-30)	30(15-45)	0.095	22 (15-45)	30(15-40)	0.611
-p27 [IRS]	12(4-12)	12(9-12)	0.565	12(4-12)	9(9-12)	0.756
-p53 [pathological, n (%)]	7(87.5)	8(88.9)	0.999	11(84.6)	4(100)	0.999
-P53(WT/overexpression 21-50%/ ≥50%, n(%))	1(12.5)/5(62.5)/2(25)	1(11.1)/3(33.3)/5(55.6)	0.420	2(15.4)/8(61.5)/3(23.1)	0(0)/0(0)/4(100)	**0.035**

Abbreviations: ACC; Adrenal cortical carcinoma, HPF; High-power fields, IRS; Immunoreactive score, WT; Wild type

Table S2. Univariate Cox Regression Analysis for Risk Factors Associated with PFS and OS in 24 Patients with ACC

	PFS		OS	
	Hazard Ratio	p-value	Hazard Ratio	p-value
**Clinical characteristics**				
Age (years)	1.04	0.154	1.02	0.558
Sex (Women)	0.38	0.149	0.51	0.407
Size (mm)	1.04	0.330	1.02	0.748
Functionality	7.02	**0.003**	5.22	**0.046**
Metastatic presentation	1.81	0.333	2.87	0.152
**Histopathological characteristics**				
Weiss (≥6)	6.34	0.078	-	-
Helsinki	1.02	0.337	1.02	0.499
Stage (3 or 4)	3.81	**0.047**	3.23	0.155
Reticulin score	0.88	0.862	0.84	0.837
**Immunohistochemical characteristics**				
Ki-67%	1.03	0.352	1.02	0.576
Capsular invasion	3.62	0.217	1.96	0.527
Vascular invasion	1.61	0.396	2.07	0.317
Mitoses >20 per 50 HPF	2.48	0.113	1.42	0.619
Atypical mitoses	1.28	0.750	-	-
P27 (IRS)	0.81	0.140	0.90	0.447
P53 (pathological)	1.39	0.757	-	-
P53 (WT/ overexpession 20-49% vs. >50%)*	3.99	**0.032**	-	-

*as continuous variable

Abbreviations: PFS; Progression free survival, OS; Overall survival, ACC; Adrenal cortical carcinoma, HPF; High-power fields, IRS; Immunoreactive score, WT; Wild type

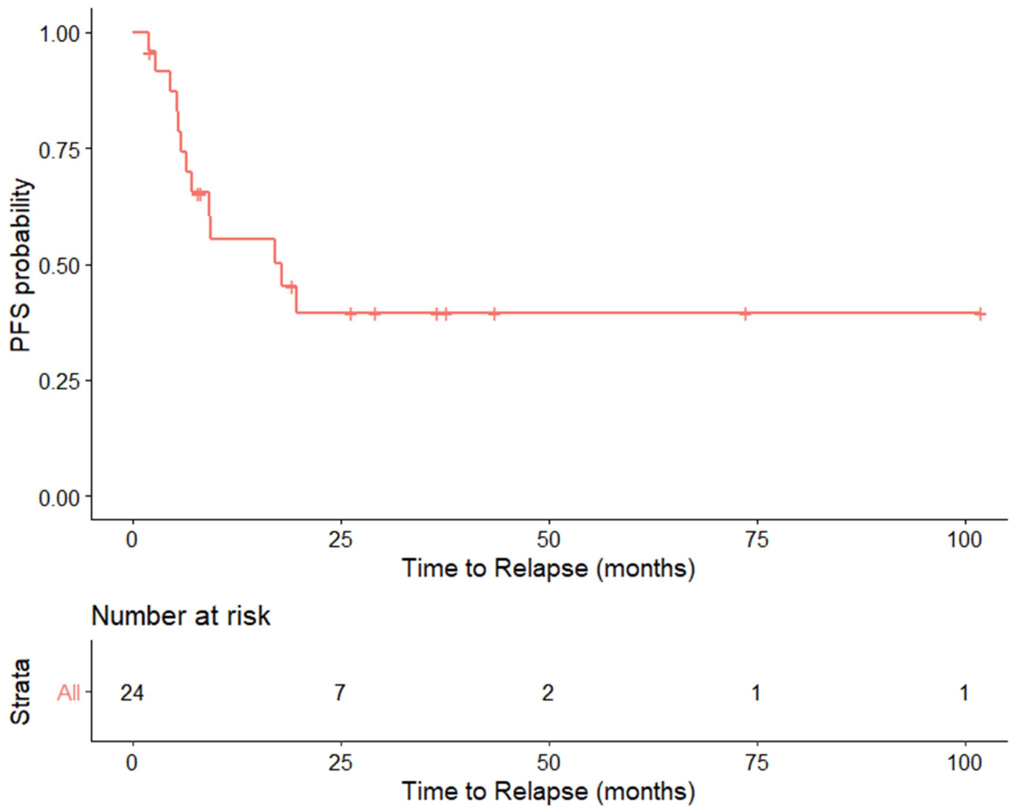

Figure S1. Kalpan Meier curve for PFS of patients with ACC

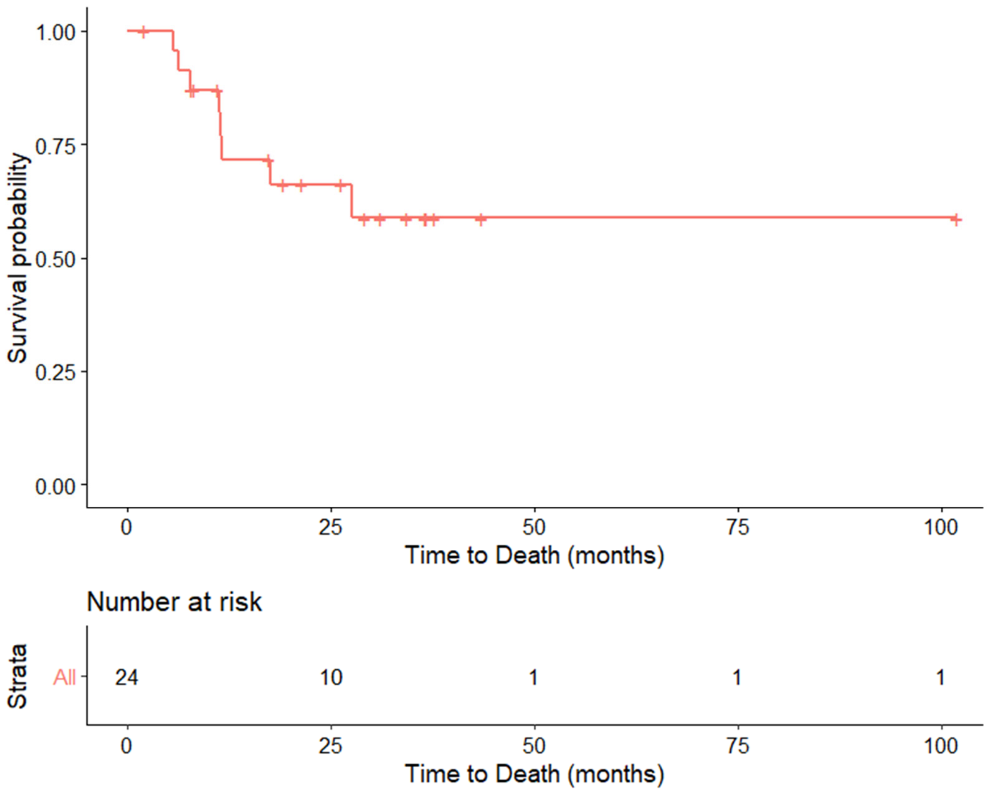

Figure S2. Kalpan Meier curve for OS of patients with ACC
